# Effects of patient movement on measurements of myocardial blood flow and viability in resting ^15^O-water PET studies

**DOI:** 10.1007/s12350-012-9522-0

**Published:** 2012-02-08

**Authors:** Kazuhiro Koshino, Hiroshi Watabe, Junichiro Enmi, Yoshiyuki Hirano, Tsutomu Zeniya, Shinji Hasegawa, Takuya Hayashi, Shigeru Miyagawa, Yoshiki Sawa, Jun Hatazawa, Hidehiro Iida

**Affiliations:** 1 Department of Investigative Radiology, National Cerebral and Cardiovascular Center Research Institute, 5-7-1 Fujishirodai, Suita, Osaka, 565-8565 Japan; 2 Department of Molecular Imaging in Medicine, Osaka University Graduate School of Medicine, Osaka, Japan; 3 Department of Cardiology, Osaka Koseinenkin Hospital, Osaka, Japan; 4 Functional Probe Research Laboratory, RIKEN Center for Molecular Imaging Science, Hyogo, Japan; 5 Department of Cardiovascular Surgery, Osaka University Graduate School of Medicine, Osaka, Japan

**Keywords:** Myocardial blood flow, water-perfusable tissue fraction, PET, myocardial perfusion imaging, motion correction, ^15^O-labeled water

## Abstract

**Background:**

Patient movement has been considered an important source of errors in cardiac PET. This study was aimed at evaluating the effects of such movement on myocardial blood flow (MBF) and perfusable tissue fraction (PTF) measurements in intravenous ^15^O-water PET.

**Methods:**

Nineteen ^15^O-water scans were performed on ten healthy volunteers and three patients with severe cardiac dysfunction under resting conditions. Motions of subjects during scans were estimated by monitoring locations of markers on their chests using an optical motion-tracking device. Each sinogram of the dynamic emission frames was corrected for subject motion. Variation of regional MBF and PTF with and without the motion corrections was evaluated.

**Results:**

In nine scans, motions during ^15^O-water scan (inter-frame (IF) motion) and misalignments relative to the transmission scan (inter-scan (IS) motion) larger than the spatial resolution of the PET scanner (4.0 mm) were both detected by the optical motion-tracking device. After correction for IF motions, MBF values changed from 0.845 ± 0.366 to 0.780 ± 0.360 mL/minute/g (*P* < .05). In four scans with only IS motion detected, PTF values changed significantly from 0.465 ± 0.118 to 0.504 ± 0.087 g/mL (*P*< .05), but no significant change was found in MBF values.

**Conclusions:**

This study demonstrates that IF motion during ^15^O-water scan at rest can be source of error in MBF measurement. Furthermore, estimated MBF is less sensitive than PTF values to misalignment between transmission and ^15^O-water emission scans.

## Introduction

Positron emission tomography (PET) has been extensively utilized for a wide range of non-invasive functional imaging of the myocardium in vivo. When using this method, the global body movements of patients could be a source of quantitative errors. Such movement could be particularly problematic when scans are carried out for a relatively long period.^1^,^2^ Problems also arise when studies are carried out during physiologically stressed conditions, e.g., a cycling exercise in the PET scanner.^3^ Errors can be attributed not only to the mismatch between the emission and the transmission data but also to the patient motion during each of the emission and/or the transmission scans.^3^

^15^O-water PET studies provide quantitative information regarding myocardial blood flow (MBF) and coronary flow reserve (CFR), as well as a marker of myocardial viability, termed the water perfusable tissue fraction (PTF) or water perfusable tissue index (PTI).^4^-^12^ The distribution of radioactivity during ^15^O-water PET varies over time; this poses challenges for software-based correction of patient movement. Naum et al proposed to correct for such motion based on the rigid body model by aligning two external radioactive markers on the back of each subject. This study was conducted by performing dynamic scans while the subjects were under resting conditions and engaged in a cycling exercise.^3^ Although no correction was made for the misalignment between transmission and emission scans, their study demonstrated reasonable improvement in calculated MBF values.

In our previous work, we developed an alternative system that uses an optical motion-tracking device to detect and correct for the patient’s global movement during a cardiac ^15^O-water PET study.^13^ Our system provides a correction for movement during dynamic scanning, as well as for misalignment between the transmission and the emission scans, to compensate for errors in attenuation correction procedures. We evaluated and methodologically validated the inherent accuracy of this system in a cardiac phantom study. The correction for simulated global movement in a ^15^O-water cardiac PET study of a healthy volunteer has also demonstrated reasonable regional MBF values, compared to values not adjusted for movement.

The purpose of this study was to evaluate the effects of global movements of subjects on quantification of MBF and PTF. Our previously validated system was used to detect and correct for the global movements of healthy volunteers, as well as patients who have suffered from severe cardiac dysfunction, during ^15^O-water PET studies under resting conditions.

## Methods

### Subjects

Subjects consisted of 10 healthy volunteers and 3 patients with previous myocardial infarction. The volunteers were all male, 22-32 years of age (mean ± 1standard deviation (SD) 25 ± 3 years). The volunteers had no signs or symptoms of ischemic heart disease. Patients were studied before and after the cell transplantation therapy with autologous myoblast sheets (AMS).^14^,^15^ Scans were carried out by independent clinical research project, but were included in this study by mutual agreement. Two of the patients were male, the other was female; patients were 43-63 years of age. All patients had left ventricular assist systems (LVASs) at the time of PET study, except for one patient who received LVAS after the first PET and before the second PET studies. The PET studies were carried out 67-104 days (mean ± SD 82 ± 19 days) after the implantation of LVASs, and 26-106 days (mean ± SD 56 ± 44 days) after AMS transplantation therapy. All subjects gave written informed consent according to a protocol approved by the Ethical Committee and Internal Review Board of Osaka University.

### PET Scan

The PET scanner was a HEADTOME-V tomograph (SHIMADZU Corp., Kyoto, Japan).^16^ All data were acquired in 2D mode. Reconstructed images were obtained using a filtered back-projection algorithm with a Gaussian filter of 9 mm (full width at half maximum). The matrix and voxel sizes of reconstructed image were 128 × 128 × 63 and 2.03 × 2.03 × 3.13 mm^3^, respectively. No scatter correction was applied to the image reconstruction.

Each subject was laid on the bed of the PET camera without any fixation of the body, and scanned at rest. A transmission scan was carried out first for correction of photon attenuation (20 minutes on the healthy volunteers, 10-15 minutes on the patients). A ^15^O-CO emission scan for blood pool imaging was initiated 8 minutes after inhalation of ^15^O-CO gas for 2 minutes (3.0-3.2 GBq). The ^15^O-water dynamic emission scan was then carried out following intravenous administration of ^15^O-water (1.1 GBq over 40 seconds) into the brachial vein, except for one patient who received the administration via right femoral vein. ^15^O-water scans were performed for 6 minutes, using 26 dynamic frames consisting of 12 × 5 s, 8 × 15 s, and 6 × 30 s. ^15^O-water scans were performed only once on eight healthy volunteers; two of the volunteers underwent ^15^O-water scans twice. Thus, a total of twelve ^15^O-water scans were carried out on the healthy volunteers. One patient underwent PET scans three times (before and after the implantation of LVAS, and after the cell transplantation therapy); the other two were scanned twice (before and after the cell transplantation therapy). Thus, a total of seven PET studies were carried out on the patients.

### Motion Detection and Correction

**Figure 1:**
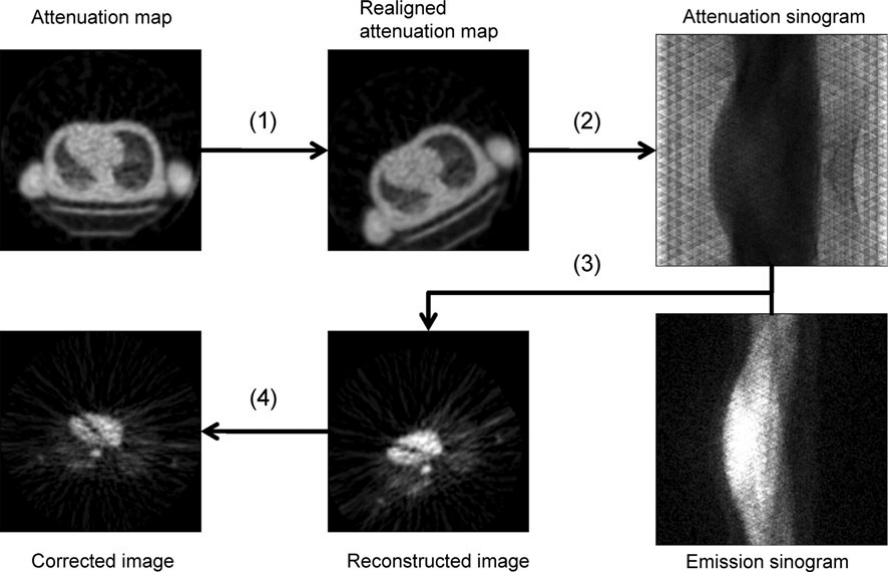
Schematic diagram of the correction for IS and IF motions

Subject motion during cardiac ^15^O-water PET was detected using an optical motion-tracking device, POLARIS (Northern Digital Inc., Canada). This method for motion correction (MC) is based on a rigid body model, and performed on each sinogram of the dynamic frame to correct for inter-scan (IS) and inter-frame (IF) motions, as shown in Figure 1. The correction process was performed automatically, based on user input consisting of subject locations measured by POLARIS, sinograms, and reconstruction parameters. In this manuscript, the IS motion denotes global motion between transmission and the first frame of ^15^O-water emission scan; the IF motion, which is in addition to the IS motion, denotes global motion between frames of the ^15^O-water scan. Methodological details regarding detection and correction of the motions are described in our previous studies.^13^,^17^,^18^

### Motion Classification

Global movement during ^15^O-water scan was characterized as consisting of IS and IF motions. Accordingly, motion was classified as: (1) IS + IF, in which both IS and IF motions were present; (2) IS motion only; (3) IF motion only; and (4) neither type of movement was present (NE). The presence of each IS and IF motion was determined as described below. Using values $t_{x} (i),\;t_{y} (i),$ and $t_{z} (i)$ to represent translational movement in the *x*, *y*, and *z*, respectively, directions between the transmission and the *i*th frame of the ^15^O-water scans, IS motion was considered significant if $\sqrt {t_{x} (1)^{2} + t_{y} (1)^{2} + t_{z} (1)^{2} } > 4.0\,{\text{mm}},$ where the value of 4.0 mm was the intrinsic spatial resolution of the PET scanner.^16^

IF motion was considered to be significant if $\mathop {\max }\limits_{i} \left\{ {L(i),H(i)} \right\} > 4.0\,{\text{mm}},$ where *L*(*i*) represents the gradual movement of the subject during the scan, and *H*(*i*) denotes the motion between adjacent frames. These two components of the IF motion can be expressed as

1a $$ L(i) = \sqrt {\sum\limits_{w = x,y,z} {\left[ {t_{w} (i) - t_{w} (1)} \right]^{2} } } $$

1b $$ H(i) = \sqrt {\sum\limits_{w = x,y,z} {\left[ {t_{w} (i) - t_{w} (i - 1)} \right]^{2} } } $$

### Motion Effect Evaluation

To evaluate motion effects on perfusion and viability measurements, we estimated regional MBF and PTF values for nine myocardial segments (anterior, lateral, posterior, and septal wall regions at middle and basal levels, as well as apex) with and without MCs, as described in the following section. Percent differences in the estimated values between with and without MCs for the nine myocardial regions were statistically tested using a one-way ANOVA to find myocardial segments sensitive to global movement. The percent differences were employed owing to avoiding difference of physiological states cross the subjects. The percent difference was defined by $\% \Updelta q(i) = 100 \times {{\left| {q_{\text{W}} (i) - q_{\text{WO}} (i)} \right|} \mathord{\left/ {\vphantom {{\left| {q_{\text{W}} (i) - q_{\text{WO}} (i)} \right|} {q_{\text{WO}} (i)}}} \right. \kern-\nulldelimiterspace} {q_{\text{WO}} (i)}},$ where *q*_W_(*i*) and *q*_WO_(*i*) are MBF or PTF values, respectively, for the *i*th myocardial segment with and without MCs. Absolute values of MBF and PTF with and without MCs for each group were also assessed using Bland-Altman analysis and a paired 2-tailed *t* test. In the IS + IF motion group, to assess the effects of IS and IF motions, additional MBF and PTF estimations were performed on the data from ^15^O-water images corrected for IS motions (IS + IF − IS) and also on data corrected for IF motions (IS + IF − IF). The quantitative values obtained from IS + IF − IS data were considered to be affected by IF motion; likewise, values obtained from the IS + IF − IF data were considered to be affected by IS motion. The estimated values were compared to those without MCs using a paired 2-tailed *t* test. *P* values <.05 were considered statistically significant. Data was expressed as mean ± 1SD.

### MBF and PTF Estimation

Regional MBF and PTF were obtained using the single tissue compartment model with correction for partial volume effects and spillover from the left ventricular cavity (LV)

$$ C(t) = {\text{MBF}} \cdot {\text{PTF}} \cdot C_{\text{a}} (t) \otimes \exp \left( { - \frac{\text{MBF}}{p}t} \right) + V_{\text{a}} \cdot C_{\text{a}} (t) $$

where *C*(*t*) represents the segment tissue time-activity curve (TTAC), *C*_a_(*t*) represents the arterial time-activity curve, *p* is the partition coefficient of water in the myocardial tissue (0.91 mL/g), and *V*_a_ is the arterial blood volume and spillover fraction from LV.^19^ TTACs were generated for the nine myocardial segments using regions of interest (ROIs) selected within each ^15^O-water myocardial image with and without MCs. The myocardial image was obtained by subtraction of the early phase of the dynamic ^15^O-water image from the later phase. The midpoint between two phases was determined for each ^15^O-water image using the contrast between myocardial regions and LV cavities. To obtain *C*_a_(*t*), a ROI was first drawn on a motion-corrected ^15^O-CO image, without regard to whether MC had been applied to the ^15^O-water image. The recovery coefficient of the LV was calculated from the ROI count on the ^15^O-CO image and the blood radioactivity concentration. Second, the ROI was transformed to the ^15^O-water image coordinate using a motion matrix that represents misalignment between transmission and the first dynamic frame of the ^15^O-water scans. This transformation was made to evaluate motion effects on the ^15^O-water scan, excluding effects of misalignment between ^15^O-CO and ^15^O-water scans. Using the transformed ROI, the LV TAC of the ^15^O-water image was calculated. Finally, *C*_a_(*t*) was derived using the LV TAC and the overall TTAC was generated from nine myocardial segments according to the previous method.^6^ To demonstrate the influence of IS motion, the ROI-based approach was not used; instead, pixel-by-pixel MBF and PTF values were estimated for a representative normal scan.^20^ To denoise and smooth these parametric images, MBF and PTF were set to zero in voxels with PTF < 0.3 g/mL or *V*_a_ > 0.8 mL/mL, and then filtered using a Gaussian filter of 14 mm (full width at half maximum).^21^ For normal scans in the group, improvement of homogeneity between myocardial segments by MC was also evaluated as $100 \times (1 - {\text{SD}}_{\text{W}} /{\text{SD}}_{\text{WO}} ),$ where SD_W_ and SD_WO_ were standard deviations of regional values with and without MCs, respectively.

## Results

Table 1 shows global movement during ^15^O-water scans, categorized by the presence of IS and IF motions. Thirteen scans (7 scans on healthy volunteers and 6 scans on patients) exhibited IS motions greater than 4.0 mm, and 10 scans (5 scans on healthy volunteers and 5 scans on patients) exhibited IF motions greater than 4.0 mm. Among these, 4 scans on healthy volunteers and 5 scans on patients showed both IS and IF motions.

**Table 1:** Characteristics of motion during ^15^O-water scans

Motion type	Total (normal) scans	IS motion (mm)	IF motion (mm)
IS + IF	9 (4)	7.7 ± 2.7	11.0 ± 4.0
IS	4 (3)	5.4 ± 1.0	2.8 ± 1.1
IF	1 (1)	2.7	5.1
NE	5 (4)	2.5 ± 0.7	1.4 ± 0.5

There was no statistically significant difference between the ROI volumes of ^15^O-water images with and without MCs for IS, IF, and NE motion groups by paired 2-tailed *t* tests, and between the volumes with and without MCs, IS + IF − IS, and IS + IF − IF motion for IS + IF motion group by a one-way ANOVA.

**Figure 2:**
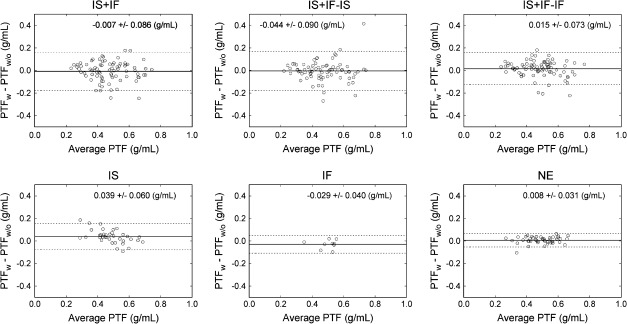
Differences between PTF values with and without the motion corrections. *IS*, Inter-scan motion; *IF*, inter-frame motion; *NE*, no significant motion. IS + IF − IS and IS + IF − IF are groups corrected for IS and IF motion in the IS + IF group, respectively

**Figure 3:**
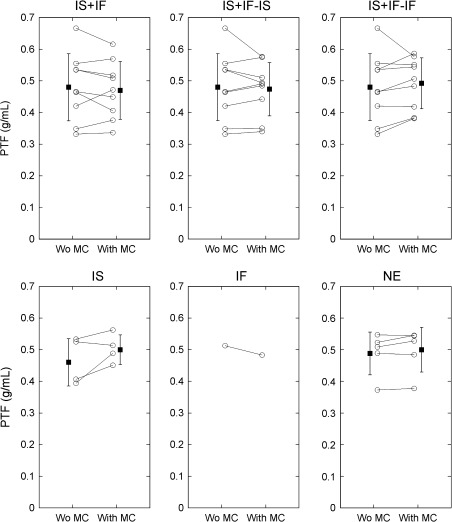
Changes in PTF values in the absolute scale by the MCs. *IS*, Inter-scan motion; *IF*, inter-frame motion; *NE*, no significant motion. IS + IF − IS and IS + IF − IF are groups corrected for IS and IF motion in the IS + IF group, respectively. A data point indicates an averaged value for each scan

Table 2 lists MBF and PTF values with and without MCs categorized by the four types of movement that occurred during ^15^O-water studies, and average percent differences of MBF and PTF values over nine segments. Among 171 myocardial segments from all subjects, the fitting program failed to provide physiologically meaningful values in two segments (middle and basal septa for a healthy volunteer, in which IS + IF motion was detected) in the absence of MC. Those data were excluded from subsequent analysis. Among MBF values, significant changes by MCs were found in the IS + IF, IS + IF − IF, and IF motion groups: for IS + IF, from 0.845 ± 0.366 to 0.769 ± 0.319 mL/minute/g (*P* < .05); for IS + IF − IF, from 0.845 ± 0.366 to 0.780 ± 0.360 mL/minute/g (*P* < .05); and for IF, from 0.854 ± 0.179 to 1.088 ± 0.154 mL/minute/g (*P* < .01). PTF values in the IS motion group changed significantly from 0.465 ± 0.118 to 0.504 ± 0.087 g/mL (*P* < .05). This significant change is also shown in Figures 2 and 3, as differences between PTF values with and without MCs. In Figure 3, a data point indicated an averaged value for each scan.

**Table 2:** The summary of MBF and PTF values with and without MCs

Motion type	Without MC	With MC	Percent difference
MBF (mL/minute/g)			
IS + IF	0.845 ± 0.366	0.769 ± 0.319*	33.8 ± 61.5
IS + IF − IS		0.815 ± 0.341	21.2 ± 38.5
IS + IF − IF		0.780 ± 0.360*	25.1 ± 41.4
IS	0.855 ± 0.343	0.828 ± 0.259	9.0 ± 8.9
IF	0.854 ± 0.179	1.088 ± 0.154^†^	30.2 ± 20.9
NE	0.898 ± 0.233	0.909 ± 0.221	9.7 ± 15.7
PTF (g/mL)			
IS + IF	0.476 ± 0.133	0.469 ± 0.128	13.9 ± 10.9
IS + IF − IS		0.471 ± 0.135	12.3 ± 13.0
IS + IF − IF		0.490 ± 0.119	12.3 ± 10.2
IS	0.465 ± 0.118	0.504 ± 0.087*	9.0 ± 9.6
IF	0.512 ± 0.067	0.483 ± 0.068	7.1 ± 5.6
NE	0.488 ± 0.095	0.496 ± 0.100	5.1 ± 4.9

*IS*, Inter-scan; *IF*, inter-frame.

NE denotes motion ≤4.0 mm. IS + IF − IS and IS + IF − IF represent IS + IF motion groups with correction of IS and IF motions, respectively.

* *P* < .05, ^†^ *P* < .01 vs without MC.

**Figure 4:**
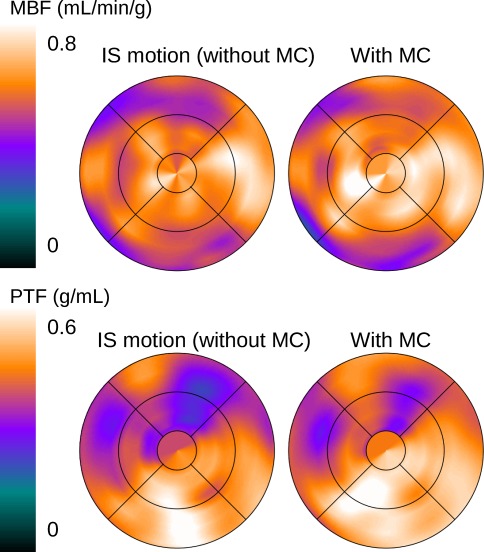
Effect of an IS motion of 5.0 mm on MBF and PTF of a normal subject in IS motion group

Figure 4 illustrates the effect of IS motion of 5.0 mm on MBF and PTF of a normal subject from the IS motion group. The polar maps of normal PTF in Figure 4 demonstrate improvement of homogeneity in anterolateral regions by MC. Improvements of homogeneity of three normal scans in the group were 2.6% ± 26.1% for MBF and 30.9% ± 22.7% for PTF.

**Figure 5:**
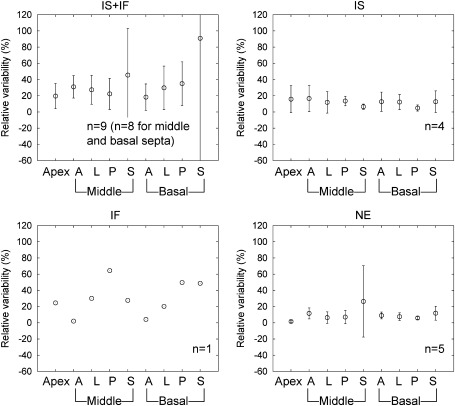
Relative variability of regional MBF due to effects of global motion. *IS*, Inter-scan motion; *IF*, inter-frame motion; NE represents no significant motion. IS + IF − IS and IS + IF − IF are groups corrected for IS and IF motion in the IS + IF group, respectively

**Figure 6:**
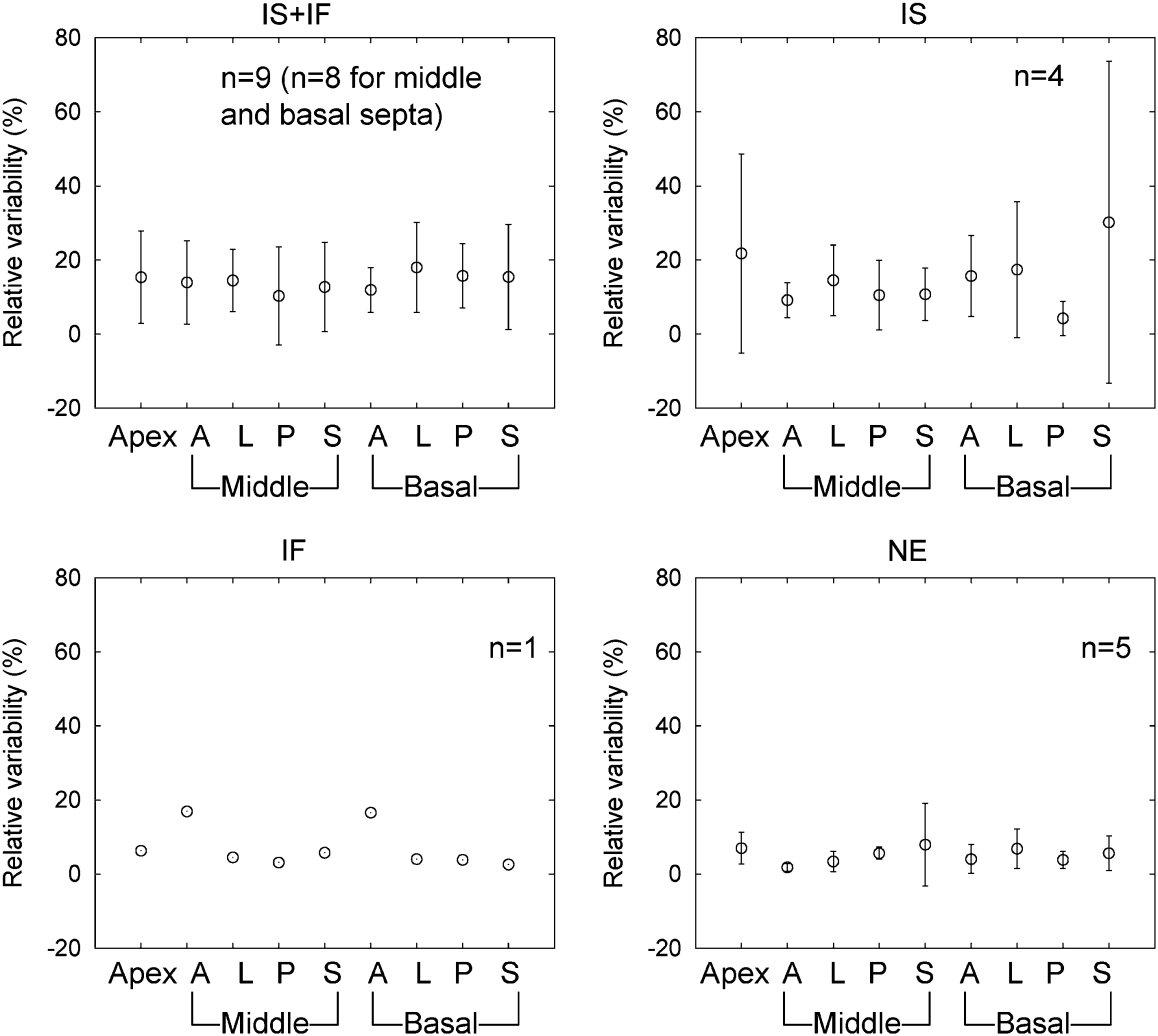
Relative variability of regional PTF due to effects of global motion. *IS*, Inter-scan motion; *IF*, inter-frame motion; NE represents no significant motion. IS + IF − IS and IS + IF − IF are groups corrected for IS and IF motion in the IS + IF group, respectively

Relative variability of regional MBF and PTF values due to global motion are shown in Figures 5 and 6, respectively. For MBF and PTF values in each motion group including the IS + IF − IS and IS + IF − IF groups, no significant difference between myocardial segments was observed in a one-way ANOVA (IF motion group was excluded from this analysis because one scan was assigned to this group. Data for IS + IF − IS and IS + IF − IF group were not shown).

## Discussion

In this study, global movements of the subjects during ^15^O-water PET scans at rest were categorized as IS and IF motions. IS motion involves misalignment relative to the transmission scan, whereas IF motions involves changes between the dynamic frames during the ^15^O-water scan. After categorization, the effects of these motions on MBF and PTF measurements were evaluated. Consequently, it was demonstrated that MBF values are affected by IF motion rather than IS motion.

Investigation of regional sensitivity to global movement resulted in no significant difference in regional MBF or PTF. This is because the direction and the magnitude of global movement varied for each subject; under- and over-estimation of MBF and PTF due to the motion could have occurred in any myocardial region. In this study, we utilized a one-way ANOVA to detect segmental differences. Due to small sample sizes, the ANOVA may fail to detect the differences. Correlation between segmental differences and the directions of global motions, especially IS motion, effects of which have been reported for misregistration between CT attenuation map and emission image,^22^,^23^ could be observed in large population study.

MBF values changed significantly upon correction for IF motions in the IF, IS + IF, and IS + IF − IF motion groups. In contrast, correction of IS motion was considered to have little effect, based on the results from the IS + IF − IS and IS motion groups in Table 2. It was further demonstrated that IF motion, but not IS motion, was an important source of error in the MBF measurement. The significant but small changes in MBF of IF, IS + IF, and IS + IF − IF motion groups after MCs could be due to the relatively large number of healthy volunteer scans in each group (1 of 1 scan for IF, 4 of 9 scans for IS + IF, respectively), as shown in Table 1. Suppression of IF motion during the ^15^O-water scan is needed for accurate MBF measurement. For the IS motion groups, no significant change in MBF values and significant change in PTF values were observed by MCs (Figures 2, 3). For the normal scans in the group, the improvement of regional homogeneity was also observed in PTF rather than MBF. This is because the MBF values obtained using the kinetic model employed in this study were based on a clearance rate assessment of ^15^O-water rather than the uptake rate of the radiotracer.^5^ This is consistent with the findings of Lubberink et al,^21^ who reported that MBF values do not change even if attenuation correction is omitted; this correction only caused changes in the absolute scale of TTAC, which then caused changes in PTF values. IS motions were also detected in the IS + IF motion group. For the IS + IF − IS motion group, in which IS motions were corrected, no significant change in PTF values was observed after MCs. This might be because IS and IF motions affected the ^15^O-water images not subjected to MC in such a way as to cancel out errors in PTF measurements. PTF values were considered to be more affected than MBF values during the wash-in phase. However, in our study, IF motion had little effect on PTF values for the IS + IF − IF and IF groups, as shown in Table 2. The reasons for this discrepancy are follows: (1) to estimate the quantitative values, a nonlinear least squares method with same weights for all data points were applied to the data corrected for the physical decay. This fitting manner could be sensitive to radioactivity concentration in wash-out phase rather than wash-in phase. (2) Over-estimation in TTAC was introduced by contamination of the LV cavity count, using the ROI for TTAC superimposed on dynamic frames. However, the spillover correction could suppress the influence of IF motion. One of the advantages of ^15^O-water over other cardiac PET tracers is that perfusion and viability measurements can be obtained from a single PET scan with short duration. Because accurate PTF measurement enabled assessment of myocardial viability,^7^-^12^ correction of IS motion, the effect of which was shown as the improvement of regional homogeneity of normal PTF in Figure 4, is considered to be important in the diagnosis of myocardial infarction, or evaluation of effects of cell transplantation therapies.

In this study, we performed ^15^O-CO scans to determine a recovery coefficient for each subject.^5^,^6^ ^15^O-water PET studies without ^15^O-CO scans have been reported in previous papers^3^,^21^,^24^; in these studies, the recovery coefficient could be fixed, or assumed to have a constant value. Omission of the ^15^O-CO scan shortened the examination time, and might reduce motion artefacts. However, we considered that the adequacy of using a fixed recovery coefficient is still an unresolved issue in ^15^O-water PET. We considered that the changes in MBF and PTF resulting from MCs were due to correction for global motion, and not due to variability of myocardial tissue ROIs drawn manually on ^15^O-water images. We employed nine segmented regions rather than 17-segment AHA standard model for suppressing relatively large noise level of ^15^O-water data.^4^ In addition, the volumes of the ROIs for each motion group before and after MCs were almost equal (not statistically significant). Furthermore, lower sensitivity of MBF values to variation in ROI size and shape was demonstrated by Iida et al.^4^ In this study, global motion might have occurred because subjects were scanned without any fixation. Although tight fixation prevents patient motion, such fixation could bring discomfort or pain to the patient; subsequent reaction to such pain could itself induce motion.

One limitation of this study is that all subjects were scanned at rest. When pharmacological or physiological stressors are administered, motion artefacts could be induced, as shown by Naum et al^3^ in the context of physiological stress conditions. Our finding that MBF was sensitive to IF motion rather than IS motion was considered to be valid for a stress study; IF motion might be source of severe error in MBF and CFR measurements. The rigid body model, which was used in this study, has been promoted for motion compensation not only in brain PET but also in cardiac PET. McCord et al^1^ employed the rigid body model to correct for misalignment during the transmission and ^18^F-fluorodeoxyglucose (^18^F-FDG) emission scans. Bacharach et al^2^ also proposed a registration technique based on the rigid body model for ^18^F-FDG emission images acquired on different days. The use of the rigid body model is considered to be valid for non-gated cardiac PET images because the images were smoothed spatially due to cardiac wall motion, and also averaged temporally over the duration of the dynamic frames. However, in stress studies, over-correction due to relatively large respiratory motions might be introduced by our system because the subject’s motion was estimated by measurement of the location of a target attached on the thoracic surface. With this in mind, further work is necessary to evaluate the contribution of MC in combination with a respiratory gating technique.

The MC system was applied to clinical follow-up studies of the patients who received LVAS and cell transplantation therapy. The aim of this study was to investigate the effects of global movement on quantification of MBF and PTF values in a single ^15^O-water PET study under resting conditions. Although, we believed that the MC system could contribute to an accurate evaluation of regional perfusion and viability, the effects of LVAS implantation and AMS transplantation therapy on those patients is beyond the scope of the present study, and will be studied elsewhere.

## Conclusions

This study demonstrated that IF motion during ^15^O-water scans under resting conditions could be the source of error in MBF measurement. Furthermore, estimated MBF values were less sensitive than PTF values to misalignment between the transmission and the ^15^O-water emission scans.
